# A Prognostic Model Incorporating Age and Systemic Inflammation Response Index for Primary CNS Lymphoma

**DOI:** 10.3390/curroncol33060345

**Published:** 2026-06-09

**Authors:** Ryosuke Matsuda, Takeshi Okuda, Hiromasa Yoshioka, Kengo Yamada, Takayuki Morimoto, Tsutomu Nakazawa, Hiromichi Hayami, Ryosuke Maeoka, Shohei Yokoyama, Ichiro Nakagawa

**Affiliations:** 1 Department of Neurosurgery, Kansai Medical University, Hirakata 573-1010, Japan; 2 Department of Neurosurgery, Nara Medical University, Kashihara 634-8522, Japan; k196441@naramed-u.ac.jp (K.Y.); t.morimoto@naramed-u.ac.jp (T.M.); nakazawa@grandsoul-immuno.co.jp (T.N.); k127245@naramed-u.ac.jp (H.H.); r.maeoka@onc.akashi.hyogo.jp (R.M.); syokoyama@naramed-u.ac.jp (S.Y.); nakagawa@naramed-u.ac.jp (I.N.); 3 Department of Neurosurgery, Kindai University Faculty of Medicine, Sakai 590-0197, Japan; okuda@med.kindai.ac.jp (T.O.); 114952@med.kindai.ac.jp (H.Y.); 4 Department of Neurosurgery, Ohnishi Neurologic Center, Akashi 674-0064, Japan

**Keywords:** primary central nervous system lymphoma, systemic inflammation response index, blood cell count, predictive scoring system

## Abstract

Background: Here, we propose a novel predictive scoring system incorporating age and the systemic inflammation response index (SIRI), which is calculated using neutrophil, monocyte, and lymphocyte counts, for patients with newly diagnosed primary central nervous system lymphoma (PCNSL). Methods: The study included 55 consecutive patients with sufficient blood test data and follow-up at our institution between November 2006 and May 2022. Age and SIRI were identified as prognostic factors and incorporated into a predictive multivariate Cox proportional hazards model. A scoring system of 0–2 points was created, with 1 point each assigned to age ≥ 65 years and high SIRI score (≥1.43 × 10^9^/L). We subsequently validated the predictive scoring system in an independent external validation cohort. Results: Patients with 0, 1, and 2 points were assigned to groups 1, 2, and 3, respectively. The median overall survival (OS) was 35.9 months in the entire training cohort and 57.8, 37.2, and 16.1 months in groups 1, 2, and 3, respectively. The three groups showed significant differences in median OS (*p* < 0.001), with lower scores corresponding to longer survival times. The performance of our new scoring system was significant in the training cohort and in the external validation cohort. Conclusion: Our new scoring system incorporating age and SIRI may serve as a preliminary prognostic model for predicting OS in patients with PCNSL. This score may be beneficial for disease risk stratification and clinical decision-making in the future.

## Simple Summary

Primary central nervous system lymphoma (PCNSL) is an aggressive brain tumor for which accurate prognostic assessment is essential for treatment planning. Existing prognostic models often require clinical or laboratory parameters that are not always readily available in routine practice. In this study, we developed and externally validated a simple prognostic scoring system based on age and the systemic inflammation response index (SIRI), a marker derived from standard blood counts. Using two independent cohorts, we demonstrated that this model effectively stratifies patients into distinct risk groups with significantly different overall survival outcomes. Importantly, the score relies only on routinely obtained laboratory data, making it highly feasible for clinical use. This preliminary prognostic model may have potential utility for future risk stratification in patients with PCNSL, although further validation is required before clinical application.

## 1. Introduction

Primary central nervous system lymphoma (PCNSL) is an aggressive extranodal non-Hodgkin lymphoma that accounts for 3–5% of all primary intracranial tumors [1,2]. Combined rituximab, high-dose methotrexate (HD-MTX), procarbazine, and vincristine (R-MPV) is one of the standard induction therapies. R-MPV combined with reduced-dose whole-brain radiotherapy (WBRT) (both with and without high-dose cytarabine) has demonstrated favorable treatment outcomes in comparison to HD-MTX with WBRT for PCNSL [3]. In addition, the MATRix regimen (combined with methotrexate, cytarabine, thiotepa, and rituximab), R-MBVP or MBVP (combined with methotrexate carmustine, teniposide, and prednisone, with and without rituximab) are promising protocols for PCNSL [4,5]. In eligible patients, autologous stem cell transplantation is one of the standard treatments for preventing recurrence and avoiding neurotoxicity caused by WBRT [6,7]. The choice of treatment from these therapeutic options is typically based on individual patient risk determined using risk prediction scores calculated from pretreatment prognostic factors. Several prognostic models have been developed to estimate outcomes in patients with PCNSL, including the Memorial Sloan–Kettering Cancer Center (MSKCC) and Extranodal Lymphoma Study Group (IELSG) scoring systems [8,9]. The IELSG model identifies five adverse prognostic factors: age greater than 60 years, an Eastern Cooperative Oncology Group performance status (ECOG PS) above 1, elevated serum lactate dehydrogenase (LDH), increased cerebrospinal fluid (CSF) protein levels, and tumor involvement of deep brain structures. The presence of these factors has been shown to correlate independently with poorer survival outcomes [8]. One of the challenges with using the IELSG score is the inclusion of pretreatment CSF protein concentration. It is not possible to perform a CSF analysis in some cases due to mass effect, particularly in a routine examination. The MSKCC score considers pretreatment age and Karnofsky performance score (KPS), based on which patients are divided into three groups to predict overall survival (OS) [9]. Recent reports have shown that adding the LDH–lymphocyte ratio to the MSKCC score results in a more accurate scoring system [10]. In fact, in the context of PCNSL, pretreatment blood test data are essential for risk prediction because inflammatory cytokines and chemokines produced by both tumor cells and stromal cells contribute to the progression of malignant tumors. In our previous report, we reported that the systemic inflammation response index (SIRI), which is calculated using the monocyte, neutrophil, and lymphocyte counts, derived from pretreatment blood test data has a significant impact on OS [11]. However, so far, no pretreatment SIRI-based scoring system for PCNSL has been reported. Accordingly, in the present study, we propose a PCNSL risk stratification system that incorporates SIRI and evaluate its usefulness in predicting OS.

## 2. Materials and Methods

### 2.1. Patient Selection for the Training and External Validation Cohorts

The medical records of patients in the training cohort were accessed from our hospital database to extract data on sex, age, pretreatment KPS, blood test results, and OS. This retrospective study was conducted with approval from the Ethics Committee of Nara Medical University (approval number 3741). We reviewed patients with newly diagnosed PCNSL who underwent treatment at Nara Medical University Hospital between November 2006 and May 2022. Histopathological confirmation of diffuse large B-cell lymphoma was required for study inclusion. A total of 58 consecutive patients were initially identified. Three patients were subsequently excluded because of insufficient pretreatment laboratory data obtained within 1 month before initial treatment (*n* = 2) or loss to follow-up (*n* = 1), leaving 55 patients eligible for analysis. Peripheral blood samples obtained before surgery or stereotactic biopsy as part of the routine pretreatment assessment were analyzed. Complete blood count data were retrieved from medical records, and several inflammation-based indices were calculated, including the neutrophil-to-lymphocyte ratio (NLR; neutrophil count/lymphocyte count), platelet-to-lymphocyte ratio (PLR; platelet count/lymphocyte count), lymphocyte-to-monocyte ratio (LMR; lymphocyte count/monocyte count), systemic immune-inflammation index (SII; platelet count × neutrophil count/lymphocyte count), and systemic inflammation response index (SIRI; neutrophil count × monocyte count/lymphocyte count). After histopathological confirmation of PCNSL, patients generally received HD-MTX-based chemotherapy as first-line treatment. Radiotherapy was administered according to physician discretion, patient condition, treatment response, and treatment era. For the external validation cohort, patient medical records from another hospital were reviewed to extract the same clinical information as that for the training cohort. The external validation cohort involves patients from entirely different regions with no overlap. This part of the protocol was approved by the Kindai University ethics committee (approval no. R07-022). Between November 2006 and March 2022, 54 consecutive patients were treated with chemotherapy and radiotherapy for PCNSL at Kindai University Hospital. Six patients lost to follow-up were excluded.

### 2.2. Statistical Analysis

OS was selected as the primary study endpoint and was measured from the date of initial surgery or stereotactic biopsy until death from any cause or the last follow-up visit. Survival distributions were estimated using the Kaplan–Meier method, and differences between groups were assessed with the log-rank test. The prognostic impact of clinical and hematological variables was evaluated, including age (≥65 vs. <65 years), sex, pretreatment KPS (≥70 vs. <70), tumor involvement of deep brain structures, number of lesions, and inflammation-based biomarkers. Variables associated with survival were first assessed using univariate Cox proportional hazards models and subsequently entered into multivariable analyses. To determine the discriminative ability of hematological markers and established clinical factors, receiver operating characteristic (ROC) curve analysis was performed. The area under the ROC curve (AUC) was calculated for each marker as well as for age and pretreatment KPS. ROC curve analysis was performed using survival status at the median overall survival time point (35.9 months) in the training cohort to determine the optimal cutoff value for SIRI. A prognostic score was generated using the independent variables identified in the multivariable Cox regression analysis. The contribution of each variable was quantified according to its regression coefficient (β), which represents the natural logarithm of the corresponding hazard ratio. To facilitate clinical application, an integer-based point system was derived by normalizing all β-coefficients to the smallest significant coefficient. Specifically, the variable with the lowest absolute β value was assigned one point, and the coefficients of the remaining variables were divided by this value to determine their relative contributions. The resulting values were rounded to the nearest whole number and used as point assignments for the scoring model. Individual patient scores were obtained by summing the points assigned to each prognostic factor. The predictive performance of the scoring system was assessed using Harrell’s concordance statistic (C-statistic). A C-statistic of 0.5 indicates no discriminative ability beyond chance, whereas a value of 1.0 reflects perfect discrimination [12]. To assess the incremental prognostic value of the proposed model, we compared C-statistics among age alone, SIRI alone, age plus KPS, and the age–SIRI model. Sensitivity analyses were performed by excluding patients who did not receive chemotherapy to evaluate whether the prognostic performance of the proposed score was influenced by treatment heterogeneity. All analyses were performed using the EZR software version 1.70 (Saitama Medical Center, Jichi Medical University, Saitama, Japan) [13], and *p* < 0.05 was considered to indicate statistical significance.

## 3. Results

### 3.1. Patient Characteristics and Survival Outcomes

The 55 patients included in the training cohort were analyzed in this study. Table 1 summarizes the patients’ characteristics in the training and external validation cohort. The training cohort included 28 men and 27 women (*n* = 55), with a median age of 68 (range 36–83) years. The mean pretreatment KPS was 63.8 ± 16.8. The involved regions of the brain were the frontal lobe (*n* = 17, 30.9%), the parietal lobe (*n* = 12, 21.8%), the basal ganglia (*n* = 16, 29.1%), the temporal lobe (*n* = 11, 20%), the cerebellum (*n* = 5, 9.1%), the occipital lobe (*n* = 4, 7.3%), the brainstem (*n* = 3, 5.5%), and the intraventricular region (*n* = 1, 1.8%). Twenty patients had multiple lesions. The mean time from blood test to surgery was 8.3 ± 5.4 days. Chemotherapy was administered in 45 of the 55 patients: HD-MTX (3 g/m^2^) in 39 patients and combined R-MPV (rituximab, 375 mg/m^2^; HD-MTX, 3 g/m^2^; procarbazine, 100 mg/m^2^; and vincristine, 1.4 mg/m^2^) in 6 patients. Intracranial MTX was administered to patients in poor general condition who had lesions detected within the ventricles. The reasons for not receiving chemotherapy were poor general condition, including advanced age in five patients, refusal of treatment in one patient, renal impairment in one patient, and unknown reasons in three patients. Radiotherapy was administered in 47 of the 55 patients. The reasons for not receiving radiotherapy were refusal of treatment in three patients, poor general condition in two patients, severe cognitive impairment in two patients, and death before initiation of radiotherapy in one patient. In the training cohort, no patients received autologous stem cell transplantation as consolidation therapy.

In the training cohort, the median follow-up time was 34.5 months (range: 1–103.2 months), and MST was 35.9 months (95% confidence interval (CI):19.7–40.8 months). In the training cohort, 50 deaths occurred during follow-up. Most deaths were considered to be related to progression of PCNSL, general deterioration associated with advanced disease, or infectious complications such as pneumonia. Other causes of death included ileus, accidental drowning associated with epileptic seizure, and cases with unknown causes of death. The reasons for not receiving chemotherapy were poor general condition, including advanced age, in five patients; refusal of treatment in one patient; renal impairment in one patient; and unknown reasons in three patients. ROC curve analysis identified a SIRI value of 1.43 as the optimal threshold for predicting OS, yielding an AUC of 0.649, a sensitivity of 74.7, and a specificity of 59.0. Kaplan–Meier survival analysis showed no significant association between OS and either sex (*p* = 0.212) or pretreatment KPS (*p* = 0.344). In contrast, significant differences in OS were observed according to age (*p* = 0.004) and SIRI status (*p* = 0.006). To further evaluate prognostic factors, Cox proportional hazards regression analyses were performed. In the univariate analysis, age younger than 65 years (HR, 2.53; 95% CI, 1.32–4.84; *p* = 0.0035) and a low SIRI value (HR, 2.33; 95% CI, 1.27–4.30; *p* = 0.006) were significantly associated with prolonged OS. These associations remained significant in the multivariable model, with age < 65 years (HR, 2.36; 95% CI, 1.21–4.58; *p* = 0.011) and low SIRI (HR, 2.07; 95% CI, 1.12–3.81; *p* = 0.019) independently predicting improved OS (Table 2).

### 3.2. New Predictive Scoring System

**Figure 1:**
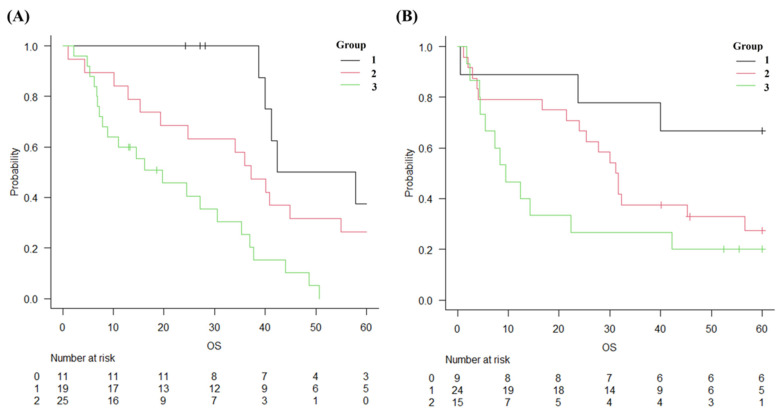
Survival analysis of the training and external validation cohorts. OS of patients with PCNSL estimated using the Kaplan–Meier method in the training cohort (**A**) and in the validation cohort (**B**).

We found that age (≥65 years vs. <65 years) and SIRI (<1.43 vs. ≥1.43) significantly affected prognosis for OS according to multivariate analysis based on pretreatment data for PCNSL. Therefore, we decided to construct a new scoring system using these two factors. To assign points in the scoring system, β-coefficients were calculated using the Cox proportional hazards model. The β-coefficient for SIRI was 0.729 and that for age was 0.858. Using the minimum value (0.729) as the reference (set to 1), the β-ratio for age was 1.18. A scoring system of 0–2 points was created, with 1 point each assigned to age ≥ 65 years and high SIRI score (≥1.43 × 10^9^/L) (Table 3). Patients were stratified into three risk categories according to their total score: group 1 (0 points), group 2 (1 point), and group 3 (2 points). In the training cohort, the median OS for the entire population was 37.0 months. When analyzed by risk group, the median OS was 57.8 months in group 1, 37.2 months in group 2, and 16.1 months in group 3. Kaplan–Meier analysis demonstrated a significant difference in survival among the three groups (*p* < 0.001), with progressively shorter survival observed as the prognostic score increased (Figure 1A). The performance of our new scoring system in predicting OS was significant in the training cohort. Kaplan–Meier estimates demonstrated clear separation of OS among the three risk groups at clinically meaningful time points. The estimated 12-, 24-, and 36-month OS rates were 100%, 100%, and 90.9% in group 1; 84.2%, 68.4%, and 52.6% in group 2; and 60.0%, 44.0%, and 28.0% in group 3, respectively. Compared with group 1, the hazard ratio for OS was 1.97 (95% CI: 0.82–4.72, *p* = 0.129) in group 2 and 4.66 (95% CI: 1.92–11.33, *p* = 0.0007) in group 3. Additionally, the C-statistic for this scoring system in the training cohort was 0.661 (95%CI: 0.59–0.73) for OS. The age–SIRI model we propose demonstrated higher discriminative ability (the C-statistic: 0.661) than age alone (0.617), SIRI alone (0.616), and the age + KPS model (0.627) in the training cohort. A supplementary calibration-style assessment at 12, 24, and 36 months in both the training and external validation cohorts demonstrated a reasonable trend between score strata and observed survival probability (Supplementary Figure S1). However, formal bootstrap-based calibration analysis was not performed because of the limited sample size.

In the external validation cohort, 35 deaths occurred. Median OS was not reached, 31.4 months, and 9.5 months in groups 1, 2, and 3, respectively. The three groups showed significant differences in the median OS (*p* = 0.0496) (Figure 1B). In the external validation cohort, the estimated 12-, 24-, and 36-month OS rates were 88.9%, 77.8%, and 77.8% in group 1; 79.2%, 70.8%, and 37.5% in group 2; and 46.7%, 26.7%, and 26.7% in group 3, respectively. Compared with group 1, the hazard ratio for OS was 2.29 (95% CI: 0.77–6.80, *p* = 0.137) in group 2 and 3.89 (95% CI: 1.22–12.33, *p* = 0.021) in group 3. Further, the C-statistic for this scoring system in the external validation cohort was 0.622 (95% CI: 0.52–0.72) for OS. In the sensitivity analysis restricted to patients who received chemotherapy, the proposed age–SIRI score showed a prognostic trend consistent with that observed in the entire cohort. The three groups showed significant differences in MST median OS (*p* = 0.0048) and the C-statistic in the sensitivity analysis in the training cohort was 0.658 (95%CI: 0.57–0.73) for OS (Supplementary Figure S2).

## 4. Discussion

PCNSL is an aggressive extranodal non-Hodgkin lymphoma that accounts for 3–5% of all primary intracranial tumors. The standard treatment for PCNSL is mainly combination therapy including MTX, and in patients receiving combination chemotherapy, 5-year survival rates have been shown to improve to a maximum of 50% to 70% [1]. In such circumstances, determining the likelihood of long-term survival and the risk of early death can provide crucial information for determining the modality and intensity of treatment before it is started. Various pretreatment predictive scoring systems have been proposed to aid with risk prediction in this context [8,9,10]. For example, the IELSG score proposed in 2003 incorporates the variables of age (≥60 vs. <60 years), ECOG PS (0–1 vs. 2–4), LDH serum level (normal vs. elevated), protein CSF concentration (normal vs. elevated), and involvement of the deep structures of the brain (no vs. yes). The total score is used to divide patients into three groups (0–1, 2–3, and 4–5 points, respectively), with low IELSG scores considered as significant and independent predictors of longer survival [8]. Another scoring system developed by researchers in Nottingham and Barcelona in 2004 considers age (cutoff = 60 years), ECOG PS (cutoff = 2), and presence of multifocal lesions or meningeal disease [14]. The MSKCC scoring system established in 2006 includes age (≥50 vs. <50 years) and pretreatment KPS and is a simple, statistically powerful model that has high universal applicability in patients with newly diagnosed PCNSL. This system restricts the predictive factors to age (≥50 vs. <50 years) and KPS only, with high weight assigned to age [9]. Some have argued that the IELSG score is not suitable in actual clinical practice as it requires the measurement of blood LDH and CSF protein, which are not routine measurements [15]. The Taipei score developed in 2019 is a new scoring system based on age, ECOG PS, and tumor location. It has the advantage of being able to predict both progression-free survival and OS. Based on its C-index, the Taipei score appears to be more accurate than both the IELSG and MSKCC scores [16]. The most recent scoring system was proposed by Ling et al., who used four markers: ECOG PS, albumin, D-dimer, and neutrophil–lymphocyte ratio (NLR). Their system makes it possible to predict 1-year, 3-year, and 5-year survival rates. However, while it shows highly sensitivity in predicting prognosis, the system consists of four items and has a total score ranging from 0 to 350 points, which requires somewhat complex calculations and reduces its clinical applicability [17]. Shin et al. proposed a prognostic model incorporating age, ECOG performance status, LDH, and serum β2-microglobulin levels in patients with PCNSL. Similar to previous models, age and performance status remained the most reproducible prognostic factors across cohorts [18].

To the best of our knowledge, there is currently no scoring system for PCNSL that utilizes SIRI. Here, we focused on blood cell counts from the patient information obtained prior to surgery. In our previous study, we reported that pretreatment SIRI is a strong predictor of OS [11]. Immune and inflammatory cells include neutrophils, monocytes, and lymphocytes, which can be detected in circulating blood and might contribute to cancer invasion and metastasis [19,20]. While there is evidence to support pretreatment NLR as a prognostic marker after chemotherapy for PCNSL [21], one study found that NLR was not a good prognostic indicator of OS after chemotherapy for PCNSL [22]. Our previous study using pretreatment blood cell counts showed that SIRI had higher sensitivity than NLR for predicting the prognosis of PCNSL after treatment [11]. Several malignancies lead to the release of myeloid growth factors, which subsequently induce an increase neutrophil production. Neutrophils are known to release a variety of pro-tumorigenic mediators, including vascular endothelial growth factor (VEGF), tumor necrosis factor (TNF), and other inflammatory cytokines. Through these mechanisms, neutrophils can promote angiogenesis and support tumor growth. Consequently, an elevated NLR may reflect a tumor-promoting inflammatory microenvironment. Nevertheless, neutrophil and lymphocyte counts can be influenced by factors unrelated to malignancy, such as acute infections or concomitant medications, which should be considered when interpreting NLR values [23]. Monocytes also play an important role in cancer biology. These cells contribute to tumor development, invasion, and metastatic spread and may differentiate into tumor-associated macrophages or myeloid-derived suppressor cells, both of which are implicated in immune evasion and tumor progression [24]. These roles and mechanisms of immune cells support the ability of pretreatment SIRI to provide more accurate prognosis prediction in PCNSL. Furthermore, it has been demonstrated that SIRI can reflect the balance between host immune and inflammatory conditions [20,25,26]. Both SII and SIRI are systemic inflammation-based biomarkers derived from routine blood counts. While SII incorporates platelet counts, SIRI includes monocyte counts, which are closely associated with tumor-associated macrophages and the immunosuppressive tumor microenvironment. In our cohort, SIRI showed superior prognostic utility and was therefore selected for the final scoring model.

The new scoring system we have proposed has the advantages of being simple and highly feasible, as it uses only age and SIRI to calculate risk scores. Furthermore, since SIRI is calculated based on neutrophil count, lymphocyte count, and monocyte count, which are included in routine blood tests, it does not require additional testing. Overall, the introduction of this new scoring system will enable simpler prediction of prognosis in patients with PCNSL and support treatment selection. The retrospective nature of this analysis might have led to inherent biases associated with such a design. Furthermore, the sample size of patients with PCNSL in each institution was small and poses an additional limitation. Finally, the lack of pretreatment blood cell count data in the excluded patients might have influenced the analysis. Unlike our previous biomarker-focused study [11], patients receiving corticosteroids or showing elevated CRP levels were not excluded in the present analysis because the purpose of this study was to establish a practical pretreatment prognostic model applicable to real-world clinical settings. Therefore, the proposed scoring system reflects prognostic stratification under routine clinical conditions rather than under strictly controlled inflammatory conditions. The discriminative ability of the present model was modest, and external validation showed only borderline statistical significance. Therefore, this scoring system should currently be considered hypothesis-generating and requires further validation in larger prospective cohorts.

## 5. Conclusions

Our new scoring system incorporating age and SIRI is a simple model that can be applied in the clinical setting to predict OS for patients with PCNSL. In the future, validation and refinement of this system may contribute to future risk stratification after further validation.

## Figures and Tables

**Table 1:** Characteristics of patients with PCNSL in the training and external validation cohorts.

Characteristic		Training Cohort	Validation Cohort
Numbers		*n* (%)	*n* (%)
		55	48
Sex			
	Female	27 (49.1%)	22 (45.8%)
	Male	28 (50.9%)	26 (54.1%)
Age (years)			
		68.0 ± 10.7	66.4 ± 12.4
	<65	18 (32.7%)	18 (37.5%)
	≥65	37 (67.3%)	30 (62.5%)
Pretreatment KPS			
	mean	63.8 ± 16.8	57.3 ± 14.5
	<70	32 (58.2%)	31 (64.6%)
	≥70	23 (41.8%)	17 (35.4%)
MSKCC			
	Age < 50 years	3 (5.5%)	4 (8.3%)
	Age ≥ 50 years and KPS ≥ 70	20 (36.4%)	16 (33.3%)
	Age ≥ 50 years and KPS < 70	32 (58.2%)	28 (58.3%)
Chemotherapy			
	HD-MTX	39 (70.9%)	37 (77.1%)
	R-MPV	5 (9.1%)	3 (6.3%)
	Others	1 (1.8%)	0 (0%)
	No	10 (18.2%)	8 (16.7%)
Radiotherapy			
	Yes	47 (85.5%)	34 (70.8%)
	No	8 (14.5%)	14 (29.2%)
Location			
	frontal lobe	17 (30.9%)	30 (62.5%)
	parietal lobe	12 (21.8%)	11 (22.9%)
	temporal lobe	11 (20%)	6 (12.5%)
	occipital lobe	4 (7.3%)	3 (6.3%)
	basal ganglia	16 (29.1%)	18 (37.5%)
	cerebellum	5 (9.1%)	12 (25%)
	brainstem	3 (5.5%)	5 (10.4%)
	dissemination	8 (14.5%)	0 (0%)
	intraventricle	1 (1.8%)	5 (10.4%)
	pituitary	0 (0%)	2 (4.2%)
Multiple lesions			
	yes	20 (36.3%)	27 (56.3%)
	no	35 (63.6%)	21 (43.8%)
Time from blood test to surgery (day)		8.3 ± 5.4	4.6 ± 4.3
SIRI			
	<1.43 × 10^9^/L	23 (41.8%)	24 (50%)
	≥1.43 × 10^9^/L	32 (58.2%)	24 (50%)

**Table 2:** The univariate and multivariate analyses for OS in the training cohort.

Variable	Univariate Analysis			Multivariate Analysis		
	HR	95%CI	*p*-Value	HR	95%CI	*p*-Value
Sex: Male	1.43	0.81–2.51	0.214			
Age ≥ 65	2.53	1.32–4.84	0.005	2.36 **	1.21–4.58 **	0.011 **
Pretreatment KPS ≤ 70	1.324	0.74–2.37	0.345			
Number of lesions	1.662	0.90–3.06	0.103			
Deep-seated lesions	1.067	0.60–1.89	0.824			
Neutrophil ≥ 4.6 × 10^9^/L	1.866	1.03–3.39	0.04	2.01 *	1.08–3.74 *	0.027 *
Lymphocyte ≤ 2.1 × 10^9^/L	1.036	0.46–2.33	0.931			
Monocyte ≥ 0.66 × 10^9^/L	1.837	0.94–3.60	0.076			
NLR ≥ 4.84	1.619	0.89–2.96	0.117			
PLR ≤ 317	2.231	0.93–5.33	0.071			
LMR ≤ 2.5	1.744	0.96–3.17	0.068			
LDH ≥ 4.84 U/L	1.567	0.85–2.90	0.151			
SII ≥ 694	2.041	1.15–3.64	0.015	1.78 *	0.99–3.22 *	0.056 *
SIRI ≥ 1.43 × 10^9^/L	2.33	1.27–4.30	0.006	2.07 *	1.13–3.81 *	0.019 *

HR: hazard ratio, CI: confidence interval, KPS: Karnofsky performance score, NLR: neutrophil–lymphocyte ratio, PLR: platelet–lymphocyte ratio, LMR: lymphocyte–monocyte ratio, SII: systemic immune-inflammation index, SIRI: systemic inflammation response index, * adjusted by age, ** adjusted by SIRI.

**Table 3:** Calculation of risk scores with the new predictive scoring system.

	β	β-Ratio	Score
SIRI ≥ 1.43	0.73	1	1
Age ≥ 65	0.86	1.18	1

Full score: 2 points.

## Data Availability

The data presented in this study are available on request from the corresponding author.

## Supplementary Materials

The following supporting information can be downloaded at https://www.mdpi.com/article/10.3390/curroncol33060345/s1. Figure S1: A supplementary calibration-style assessment at 12, 24, and 36 months in both the training cohort; Figure S2: The sensitivity analysis restricted to patients who received chemotherapy.

## Abbreviations

The following abbreviations are used in this manuscript:

SIRI	Systemic inflammation response index
PCNSL	Primary central nervous system lymphoma
HD-MTX	High-dose methotrexate
R-MPV	Rituximab, high-dose methotrexate, procarbazine, and vincristine
WBRT	Whole-brain radiotherapy
IELSG	The International Extranodal Lymphoma Study Group
MSKCC	The Memorial Sloan–Kettering Cancer Center
ECOG PS	Eastern Cooperative Oncology Group performance status
LDH	Lactate dehydrogenase
CSF	Cerebrospinal fluid
KPS	Karnofsky performance score
OS	Overall survival
ROC	Receiver operating characteristic
AUC	Area under the curve
CI	Confidence interval
NLR	Neutrophil–lymphocyte ratio
