# Supplementary material for: A Prognostic Model Incorporating Age and Systemic Inflammation Response Index for Primary CNS Lymphoma
Source: Curr Oncol. 2026 Jun 9;33(6):345. doi: 10.3390/curroncol33060345 (PMC13297882; doi:10.3390/curroncol33060345)
Supplement: Supplementary file 1 [file curroncol-33-00345-s001.zip › curroncol-4304891-supplementary-title added/curroncol-4304891-supplementary.pptx]

## Slide 1
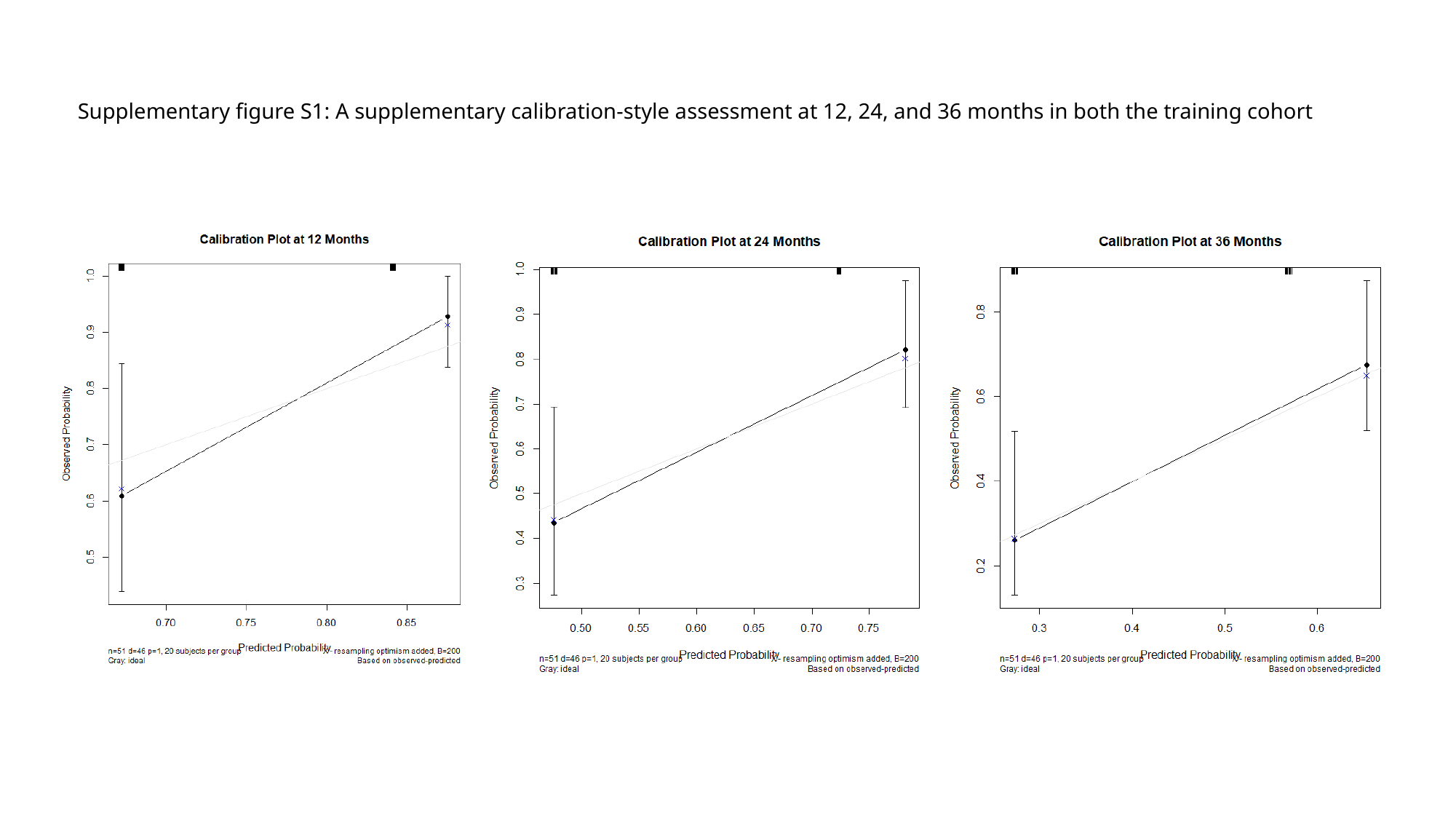

Supplementary figure S1: A supplementary calibration-style assessment at 12, 24, and 36 months in both the training cohort

## Slide 2
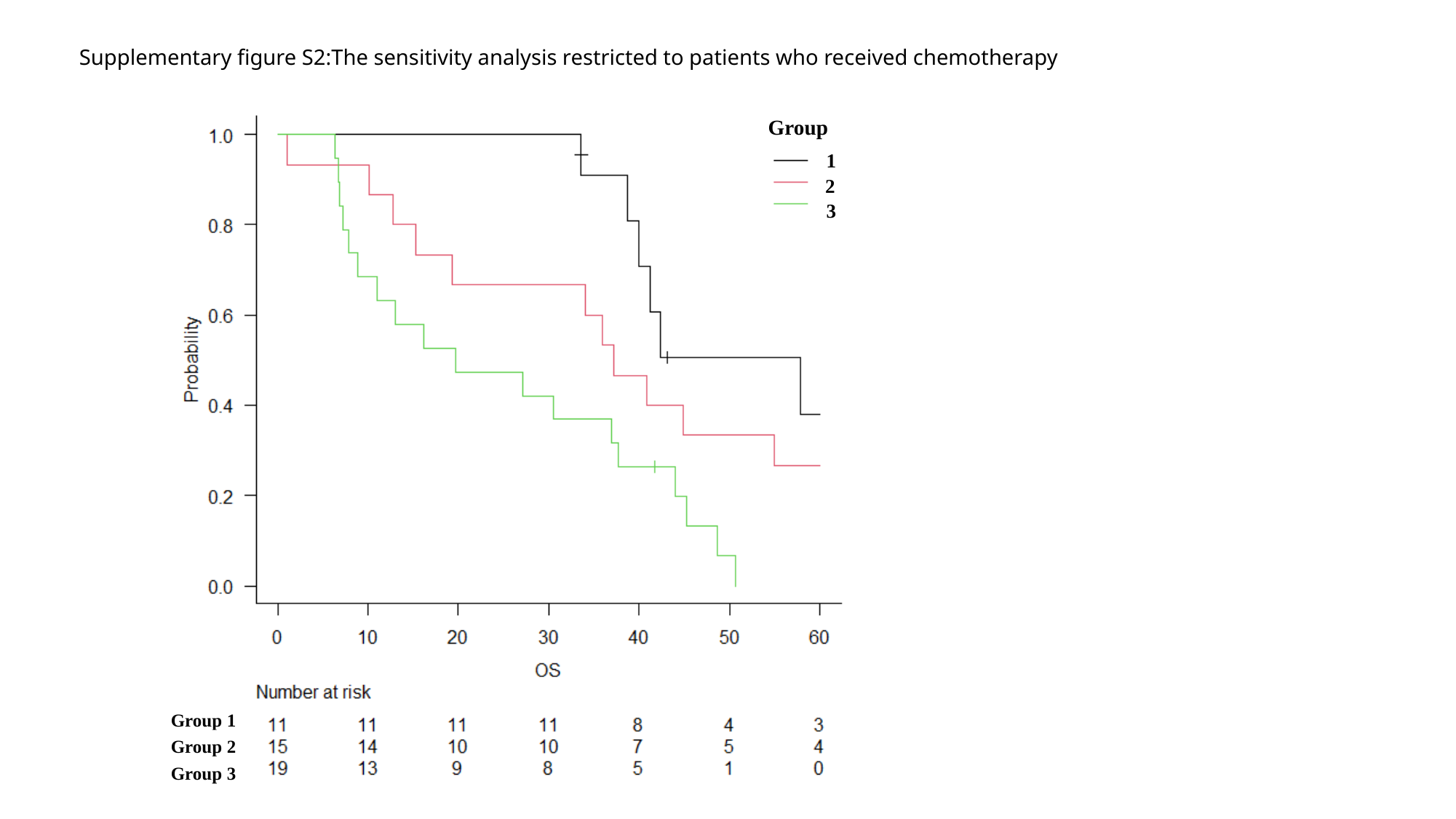

Group
1
2
3
Group 1
Group 2
Group 3
Supplementary figure S2:The sensitivity analysis restricted to patients who received chemotherapy
